# An overview of secretion in floral bracts of Tillandsioideae (Bromeliaceae), with emphasis on the secretory scales

**DOI:** 10.1093/aobpla/plad066

**Published:** 2023-09-26

**Authors:** Igor Ballego-Campos, Rafaela C Forzza, Élder A S Paiva

**Affiliations:** Departamento de Botânica, Instituto de Ciências Biológicas, Universidade Federal de Minas Gerais, Belo Horizonte, 31270-901, Minas Gerais, Brazil; Jardim Botânico do Rio de Janeiro, 22460-030 Rio de Janeiro, Brazil; Departamento de Botânica, Instituto de Ciências Biológicas, Universidade Federal de Minas Gerais, Belo Horizonte, 31270-901, Minas Gerais, Brazil

**Keywords:** Bromeliaceae, colleters, desiccation, mucilage, plant–environment interactions, plant secretion, resin, *Tillandsia*, Tillandsioideae, *Vriesea*

## Abstract

Bromeliaceae display many water-use strategies, from leaf impounding tanks to Crassulacean acid metabolism (CAM) photosynthesis and absorbing trichomes. Recent studies show that trichomes in inflorescences of bromeliads can exude viscous secretions, protecting against various stresses, including excessive water loss. In light of this, and considering the knowledge gap regarding inflorescence trichomes in bromeliads, we aimed to investigate the presence, source and chemical nature of inflorescence secretions in species of the Tillandsioideae (Bromeliaceae) and to describe the anatomy of their floral bracts focusing on trichome structure and position. We conducted a prospection of secretory activity and anatomy in floral bracts in 52 species of Tillandsioideae and 1 early divergent Bromeliaceae species. We used histochemical tests to investigate the presence and nature of secretion combined with standard light microscopy methods. Secretion appears in all studied species of tribe Vrieseeae, in *Guzmania* species, *Wallisia cyanea*, *Tillandsia streptopylla* (Tillandsieae) and *Catopsis morreniana* (Catopsideae). It is absent in *Vriesea guttata* (Vrieseeae), *Racinaea crispa* and various *Tillandsia* species (Tillandsieae). Secretion is produced by peltate trichomes on the adaxial surface of young bracts and comprises hydrophilic and lipophilic substances. Bract anatomy revealed an internal mucilage-secreting tissue with wide distribution within the subtribe Vrieseinae. Our results point to a broad occurrence of secretion associated with bracteal scales in inflorescences of Tillandsioideae. Secretory function is strongly related to trichomes of the adaxial surface, whereas the indumentum of the abaxial side is lacking or likely associated with water absorption; the latter case is especially related to small, xeric plants. Exudates might engage in colleter-like roles, protecting against desiccation, high-radiation and herbivores. Directions for future research are presented.

## Teaser

Peltate trichomes are a typical feature of bromeliads. These scale-like appendages account for the establishment of some of the most extreme epiphytic life forms among vascular plants. The investigation of their secretory function in the floral bracts of several bromeliad species revealed that their newly discovered ability to synthesize and release mucilage, oils or resins is an overlooked phenomenon that might be widespread. While further studies are needed, the structure and position of the trichomes, the chemical nature of their secretion and other anatomical features of the bracts produce new insights into the ecology, evolution and systematics of bromeliads.

## Introduction

Water is the major limiting resource for growth in land plants (Xiong and Nadal 2020). It is required in large quantities and is critical for most physiological processes, from the transport of nutrients and metabolites to mechanical support in herbaceous plants (Lambers and Oliveira 2019). Accordingly, strategies to cope with water-related stresses have been discussed profusely in terrestrial plants, both on physiological and structural grounds (Aroca 2012). In Bromeliaceae, the biology of adaptations related to water-use strategies is extremely diverse, including not only leaf impounding tanks but also the mesophyll structure, photosynthetic pathways and trichomes (Benzing 2000; Males 2016; Nogueira *et al*. 2019, 2021). These adaptations, in turn, are related to the occupation of diverse niches, including epiphytic and lithophytic life forms, through an extensive process of adaptive radiation (Benzing 2000; Givnish *et al.* 2014; Males 2016).

Among these adaptative features, the typical peltate trichomes of bromeliads, also called scales, are considered a key trait due to their capacity for efficient water absorption (Benzing 2000; Givnish 2014). Together with other features, these absorbing scales account for variable independence from functional roots, leading to extreme atmospheric life forms that have expanded bromeliads adaptive zones beyond other known epiphytes (Givnish 2014). Not surprisingly, most of the studies on bromeliad trichomes deal with their absorptive capacity. Also, these studies are often restricted to the investigation of the vegetative organs, especially leaves of atmospheric *Tillandsia* species and water-impounding taxa. Trichomes of the reproductive organs, in turn, are much less understood.

Trichomes on the inflorescence of certain bromeliads were suggested to produce visual signs for pollinators and dispersers (Benzing 2000), but little is known about their significance beyond this putative function. Carvalho *et al.* (2017) showed extreme polymorphism in floral peltate trichomes of three *Dyckia* species and found this character valuable for species delimitation. Recent findings showed that the mucilage covering the young inflorescences of *Aechmea blanchetiana* and *Wallisia cyanea* is produced and exuded by the peltate trichomes, and is possibly involved in mechanisms to cope with water-related stresses (Ballego-Campos and Paiva 2018; Ballego-Campos *et al.* 2020).

In bromeliads, the presence of secretion covering the inflorescence has been long known from anecdotal observations or species characterization in herbarium material and taxonomic studies (Benzing 2000; Moura 2011; Moura and Costa 2014; Gomes-da-Silva and Souza-Chies 2018). The presence of an oily secretion, for instance, is reported for many *Vriesea* species, and it was demonstrated that this secretion acts as a sticky trap for herbivores, effectively reducing damage in *Vriesea bituminosa* (Monteiro and Macedo 2014). Also, a study with three *Vriesea* species recently demonstrated that the scales in floral bracts can produce these oily secretions (Ballego-Campos *et al.* 2023). These findings provide new insight into the functional biology of bromeliad trichomes and call for a broader investigation of scales in the reproductive axis of Bromeliaceae.

Tillandsioideae is the largest of the eight subfamilies currently accepted for Bromeliaceae (Barfuss *et al.* 2016). This subfamily comprises a morphologically diverse group of plants, counting over 1500 species (Smith and Downs 1977; Givnish *et al*. 2011; Barfuss *et al*. 2016; Gouda and Butcher 2023). The phylogenetic relationships within the subfamily have received great attention recently (Barfuss *et al.* 2016; Gomes-da-Silva and Souza-Chies 2018; Machado *et al.* 2020). Based on molecular and morphological data, species of Tillandsioideae were recovered with good support in four distinctive tribes: Tillandsieae, Vrieseeae, Catopsideae and Glomeropticairnieae (Barfuss *et al.* 2016; Gomes-da-Silva and Souza-Chies 2018; Machado *et al*. 2020). Among these, Tillandsieae and Vrieseeae comprise the core group, with most species. Vrieseeae, in turn, splits into two major lineages generally ascribed to eastern Brazil (subtribe Vrieseinae) and the Andes, Central America and the Caribbean (subtribe Cipuropsidineae; Barfuss, *et al*. 2016; but see Machado *et al*. 2020). While the phylogenetic relationships in Tillandsioideae have seen great advancement in recent years (Barfuss *et al*. 2016; Gomes-da-Silva and Souza-Chies 2018; Machado *et al*. 2020), generic and infrageneric systematics are still in need of further resolution (Barfuss *et al*. 2016; Machado *et al*. 2020).

In light of the little knowledge regarding trichomes in inflorescences of bromeliads, as well as the recent discoveries regarding secretion in inflorescences of Tillandsioideae species (Ballego-Campos *et al.* 2020; Ballego-Campos *et al*. 2023), in this study, we investigated the presence of secretion in the floral bracts of 52 species of Tillandsioideae and 1 early-divergent bromeliad species, *Brocchinia reducta* Baker (Brocchinioideae). Our main goals were to investigate the presence and source of secretion in inflorescences, including the general nature of the exudate, and to describe the anatomy of floral bracts in the studied species, focusing on trichome structure and position. We specifically tested the following hypotheses: (a) secretion by inflorescence bracts is widespread within Tillandsioideae, potentially comprising a synapomorphy of the subfamily; (b) secretion, when present, is produced by peltate trichomes; (c) the secretions are mainly mucilaginous in nature, the presence of lipophilic secretions in *Vriesea* species (Ballego-Campos *et al*. 2023) being a derived trait of members within *Vriesea*. sect. Xiphion.

## Material and Methods

### Sampling and plant material

Plant material was obtained during multiple flowering episodes over the years 2017–2020, from material growing in the living collections of the Jardim Botânico do Rio de Janeiro (RBvb; Rio de Janeiro, Brazil, 22°58ʹ02.1″S 43°13ʹ42.3″W) and Jardim Botânico de Belo Horizonte (CPVJBBH; Minas Gerais, Brazil, 19°51ʹ30.5″S 44°00ʹ42.3″W). Additionally, *Tillandsia loliaceae* and *T*. *recurvata* specimens were collected from natural populations growing on the campus of the Universidade Federal de Minas Gerais (Belo Horizonte, Brazil, 19°52ʹ14.1″S 43°58ʹ02.3″W). We sampled 52 species of Tillandsioideae (12 genera) and one early-divergent bromeliad species, *B. reducta* (Brocchinioideae; Table 1). Nomenclature and phylogenetical inferences followed Barfuss *et al.* (2016) and Machado *et al.* (2020). The taxonomic revision proposed by Barfuss *et al.* (2016) has received some criticism (Gomes-da-Silva and Souza-Chies 2018), but recent advancements have greatly supported this revision (Machado *et al.* 2020). The adoption of this new approach includes, among other changes, the replacement of *Tillandsia cyanea* Linden ex K. Koch in a new combination within *Wallisia* (Tillandsieae: Tillandsioideae), as *W. cyanea* Barfuss & W. Till (Table 1).

**Table 1.** Presence of trichomes, secretion and mucilaginous tissue in the floral bracts of the studied species.

Taxa	Trichomes		Secretion		Mucilaginous tissue	Voucher specimens
	Presence	Type	Mucilage	Lipids		
**Tillandsioideae subfamily**						
* * **Catopsideae tribe**						
* Catopsis*						
* C. morreniana*	**AD**/AB	B*	+	–	–	CVJBFZB 387
** Tillandsieae tribe**						
* Guzmania*						
* G.* aff*. strobilifera*	**AD**/AB	Various	+	–	–	CVJBFZB 283;
* G. patula*	**AD**/AB	Various	+	–	–	CVJBFZB 3470;
* G. rhonhofiana*	**AD**/AB	Various	+	–	–	CVJBFZB 2857;
* G. roezlii* (E. Morren)	**AD**/AB	Various	+	–	–	CVJBFZB 589; BHZB 1147
* G. sprucei* (André)	**AD**/AB	Various	+	–	–	CVJBFZB 388; BHZB 11081
* G. wittmackii* (André)	**AD**/AB	Various	+	–	–	CVJBFZB 237, 324, 2880; BHZB 11169
* Racinaea*						
* R. crispa* (Baker) M.A.	AD/**AB**	Various*	–	–	–	BHCB 214565
* Tillandsia*						
* T. araujei*	–	C	–	–	–	RBvb 2310
* T. loliacea*	AB	C	–	–	–	BHCB 214283
* T. mallemontii*	AB	C	–	–	–	CVJBFZB 3370
* T. recurvata*	AB	C	–	–	–	BHCB 214281
* T. streptophylla*	AD/**AB**	C	+	–	–	RB 802224
* T. stricta*	–	C	–	–	–	RB 802223
* T. tenuifolia*	–	C	–	–	–	CVJBFZB 1194, 1249
* Wallisia*						
*W. cyanea*	**AD**/AB	A*	+	–	–	BHZB 4713; BHZB 13215; BHZB 13216
* * **Vrieseeae tribe**						
* * **Cipuropsidinae subtribe**						
* Cipuropsis-Mezobromelia* complex						
* M. pleiosticha*	**AD**/AB	B	+	**–**	+	CVJBFZB 435
* V.* aff*. rubra*	**AD**/AB	B	+	**–**	–	CVJBFZB 557
* Goudea*						
* Go. chrysostachys*	AD	B	+	–	–	CVJBFZB 262, 542
* G. ospinae*	**AD**/AB	A	+	–	–	CVJBFZB 2858
* Werauhia*						
* W. viridiflora*	AD/**AB**	B	+	–	–	CVJBFZB 130
* * **Vrieseinae subtribe**						
* Alcantarea*						
* A. burle-marxii*	AD	A	+	+	+	RB 596781, RBvb 2
* A. compacta*	AD	A	+	–	–	CVJBFZB 3085
* A. extensa*	AD	A	+	+	+	CVJBFZB 1160
* A. farneyi*	AD	A*	+	–	–	CVJBFZB 710
* A. pataxoana*	AD	A	+	+	+	RB 342724, 596680; RBvb 5
* Stigmatodon*						
* S. goniorachis*	AD	A	+	+	+	RBvb 1785
* Vriesea*						
* V.* aff*. bituminosa*	AD	B	+	+	+	CVJBFZB 1111, 1151, 1452
* V. botafogensis*	AD	A	+	+	+	RBvb 2168
* V. brusquensis*	**AD**/AB	A*	+	–	+	CVJBFZB 180
* V. carinata*	**AD**/AB	A	+	–	+	RBvb 266, 257, 2450; RB342436, 790354, 329084
* V. erythrodactylon*	**AD**/AB	A*	+	–	+	CVJBFZB 220, 3457
* V. fenestralis*	AD	A	+	+	+	CVJBFZB 713
* V. flammea*	**AD**/AB	A*	+	–	+	CVJBFZB 3
* V. flava*	AD	A	+	–	+	CVJBFZB 496
* V. friburgensis*	AD	A	+	–	+	CVJBFZB 496
* V. guttata*	AD	A	–	–	+	CVJBFZB 536
* V. incurvata*	**AD**/AB	A	+	–	+	CVJBFZB 197, 2910; RBvb 560; RB 342547
* V. lubbersii* (Baker)	AD	A	+	–	+	CVJBFZB 2858; BHZB 7891
* V. minarum*	AD	A	+	+	+	CVJBFZB 1162, 1215; BHZB 6487
* V. paraibica*	AD	A	+	–	+	RBvb 68; RB 531299
* V. pinottii*	**AD**/AB	A	+	–	+	CVJBFZB 11
* V. platynema*	AD	A	+	–	+	CVJBFZB 148, 535
* V. platynema* var*. rosea*	AD	A	+	+	+	CVJBFZB 593
* V. poenulata* (Baker)	AD/**AB**	C	+	–	+	RBvb 2473
* V. procera*	AD	A	+	–	+	RBvb 1036, RB 462661
* V. psittacina*	**AD**/AB	A*	+	–	+	CVJBFZB 252
* V. ruschii*	AD	A	+	–	+	CVJBFZB 321
* V. scalaris*	**AD**/AB	A	+	–	+	CVJBFZB 6357; RBvb 637, 395, 396; RB 531298
* V. simplex*	AD	A	+	–	+	RBvb 72, RB 439163
* V. stricta*	AD	B	+	+	+	CVJBFZB 3085
* V. warmingii*	AD	A	+	–	+	CVJBFZB1097
**Brocchinioideae subfamily** (outgroup)						
* Brocchinia*						
* B. reducta*	rarely seen	N/A	–	–	–	RBvb 899, RB 568168

AB, abaxial; AD, adaxial surface; bold indicate predominant presence; +, present: –, absent; * distinct with one or more type C-features. For trichome types (A, B and C), see Table 2.

Samples were collected from inflorescences at an early stage of flowering and divided into two categories, following Ballego-Campos *et al.* (2020): (i) young portions (with less than 50% of expansion) and (ii) mature portions (with 50% or more of total expansion). Based on the acropetally maturation of the floral parts, samples were often obtained from the same inflorescence, in which case young portions comprised the apical third of the inflorescence and mature portions comprised the middle to basal inflorescence (Ballego-Campos *et al.* 2020). In ramified inflorescences, this categorization was applied to each ramification individually.

### Fresh-material prospecting

Inflorescences at an early stage of flowering were screened for the presence of secretion both in the field and in the laboratory with a stereomicroscope (M205 C, Leica Microsystems Inc., Deerfield, USA). We searched for the presence of secretion outside the overall inflorescence and in the interior (adaxial face) of floral bracts, recording the aspect (viscosity, colour, presence/absence of scent), and overall abundancy (abundant, scarce, absent/indetectable). Additionally, secretion drops were deposited onto glass slides and left to dry; we observed whether this secretion changed viscosity over time and if residual material, such as solid films and gums, was left on the slide. The dried product was also stained with Ruthenium Red (0.002%, aqueous solution, Johansen 1940) and Alcian Blue (Pearse 1980) to detect the presence of mucilage, and Sudan Red 7B (modified from Brundrett *et al*. 1991) for lipids.

To further corroborate the secretion and to investigate the role of trichomes in the secretory process, the adaxial surface of young and mature floral bracts (at least three per maturation phase, per individual) were carefully scrapped (Ballego-Campos *et al*. 2020). The material obtained (i.e. trichomes and secretion) was placed onto glass slides and tested using Ruthenium Red, Alcian Blue and Sudan Red 7B (see above). To avoid loss of the scraped material, the glass slides were mounted using the corresponding histochemical reagent, which was then substituted with distilled water with the aid of filter paper strips and a plastic pipette. Times and protocols for histochemical tests followed Pearse (1980); Brundrett *et al*. (1991); Figueiredo *et al.* (2007); and Ventrella *et al.* (2013). Control tests were performed accordingly (see Figueiredo *et al.* 2007; Ventrella *et al.* 2013) simultaneously, using the same scrapped material from bracts.

### Light microscopy

Fresh samples of young and mature floral bracts (middle third portion, three to five per individual, per maturation stage) were obtained, fixed under a slight vacuum in Karnovsky solution (pH 7.2 in 0.1 M phosphate buffer; modified from Karnovsky 1965) for 24 h, dehydrated in an increasing ethanol series and embedded in synthetic resin (2-hydroxyethyl methacrylate, Leica®, Heidelberg, German). Transversal sections (6–8 µm) were obtained using a rotary microtome (Hyrax M40, Carl Zeiss Mikroskopie, Jena, Germany), placed onto glass slides, and stained with Toluidine Blue O (pH 4.7 in acetate buffer, modified from O’Brien *et al.* 1964) and then counterstained with Ruthenium Red (aqueous solution, 0.002%; Ballego-Campos *et al.* 2023). Slides were then mounted with synthetic resin (Entellan®, Sigma-Aldrich, St. Louis, USA) and observed under a light microscope (Olympus Scientific Solutions, Waltham, USA) equipped with a camera and an image capture software (TV0.5XC-3, Olympus Scientific Solutions, Waltham, USA). Additionally, unstained sections were subjected to histochemical tests using Ruthenium Red and Sudan Red 7B (modified from Brundrett *et al*. 1991). Control tests were performed accordingly.

## Results

### Secretion presence and appearance

**Figure 1.**
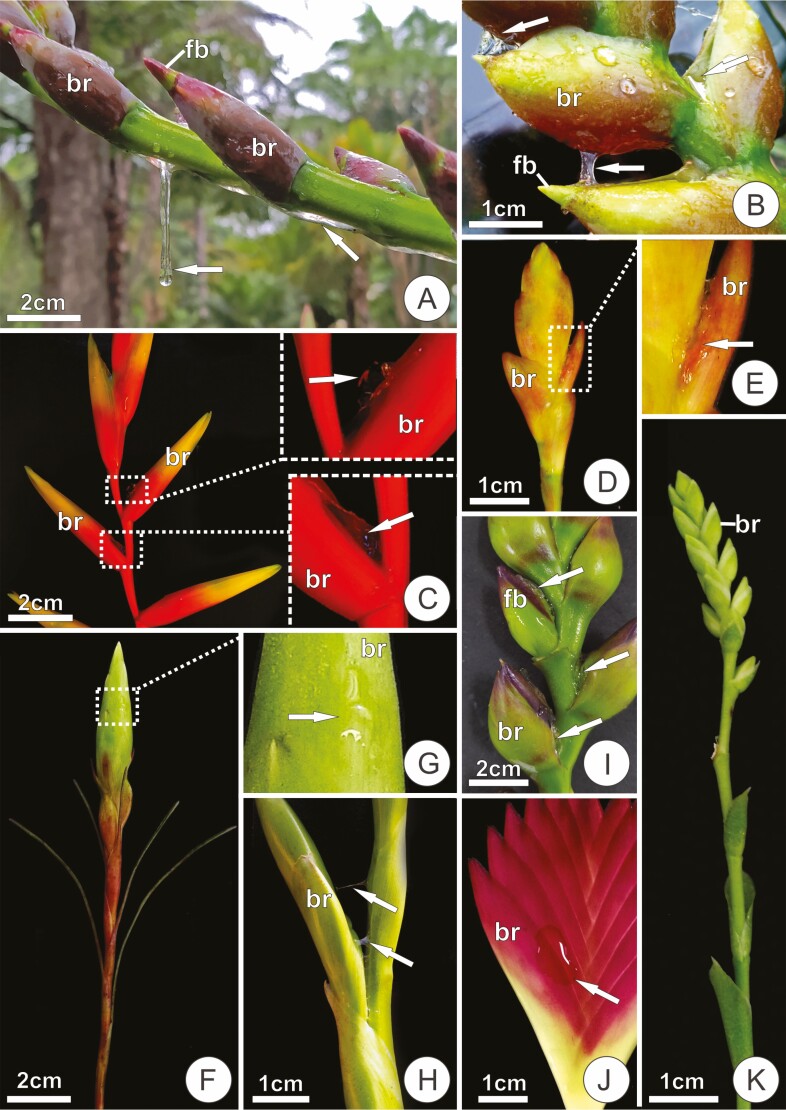
Secretion in inflorescences of some Tillandsioideae species. (**A and B**) *Alcantarea extensa*. (**A**) Lateral ramification of an inflorescence with a viscous, clear secretion dripping off the inflorescence (arrows). (**B**) Young portion displaying secretion (arrows) associated with floral bracts. (**C)***Vriesea simplex* with secretion accumulated inside the bracts (arrows, visible prior to manipulation). (**D and E**) *Vriesa pinottii*. (**D**) Lateral ramification of an inflorescence in an early stage of expansion. (**E**) Detail of secretion associated with a floral bract. (**F and G**) *Vriesea poenulata*. Young inflorescence with secretion (in G, arrow) appearing after manipulation. (**H**) *Vriesa stricta*. Portion of a young inflorescence. Note the strands of sticky secretion (arrows). (**I**) *Stigmatodon goniorrachis*. Portion of an inflorescence with secretion accumulated inside the bracts and around an exposed flower bud (fb; associated bract removed). (**J**) Inflorescence of *W. cyanea* with secretion appearing after manipulation (arrow). (**K**) Young inflorescence of *Catopsis morrenniana* (lateral ramifications removed), in which secretion was not observed. br, flower bract; fb, floral bud.

Within tribe Vrieseeae, all studied species displayed a secretion that accumulates on the adaxial surface of floral bracts, except for *V. guttata*, in which no exudate could be observed (Table 1). The exudate abundance and aspect varied between and within genera. In *Alcantarea*, secretion was seen in all studied species (Table 1) as a clear, gelatinous and remarkably abundant exudate, especially in *A. burle-marxii*, *A. extensa, A. farneyi* and *A. pataxoana* (Fig. 1A and B). In *A. extensa*, secretion not only accumulated in the bracts but sometimes dripped off the inflorescence (Fig. 1A). Likewise, in most *Vriesea* spp., secretion was conspicuous; it could be seen accumulating in the interior of bracts, but it was usually retained within the spaces between the bracts and the inflorescence axis (Fig. 1C–E), sometimes appearing only after manipulation of the inflorescence (Fig. 1F and G). The secretion in *Vriesea* species was usually a clear, gelatinous exudate; but in *V.* aff. *bituminosa, V. botafoguensis, V. fenestralis, V. friburguensis, V. minarum, V. platynema*, *V. procera, V. ruschii* and *V*. *stricta* the exudate was particularly viscous, oily and sticky (Fig. 1H). This exudate assumed a yellowish tone in *V.* aff*. bituminosa*, *V. fenestralis*, *V. ruschii* and *V. stricta*. In *Stigmatodon goniorachis*, secretion was abundant, viscous and dense, with an oily aspect in young unexposed parts, but taking a more gelatinous appearance in mature exposed bracts with increased volumes of secretion (Fig. 1I). *Goudea chrysostachys*, *G. ospinae* and *Werauhia viridiflora* also exhibited exudation of a clear, viscous secretion in the adaxial side of the bracts. In contrast, the two studied species of the *Cipuropsis-Mezobromelia* complex showed little secretion, with a very fluid aspect, only perceptible in young bracts. The presence of a distinct scent was noted only in *V.* aff. *bituminosa*, *V. fenestralis* and *V. platynema* var. *rosea* (see also Ballego-Campos *et al.* 2023).

In species of Tillandsieae, a clear secretion was observed on the adaxial surface of bracts only in *W. cyanea* (Fig. 1J) and all studied *Guzmania* species (Table 1). In the former, the exudate was similar to the secretion of many *Vriesea* species, while in members of *Guzmania*, secretion was scarce and often difficult to distinguish from accumulated water due to its less viscosity. *Racinaea crispa* and most of the *Tillandsia* species did not show signs of accumulated secretion on the adaxial surface of floral bracts (Table 1). The same was observed for inflorescences of *B. reducta* and *Catopsis morreniana* (Table 1), in which the floral bracts always appeared dry, with no apparent secretion (Fig. 1K).

### Trichome presence and position

**Figure 2.**
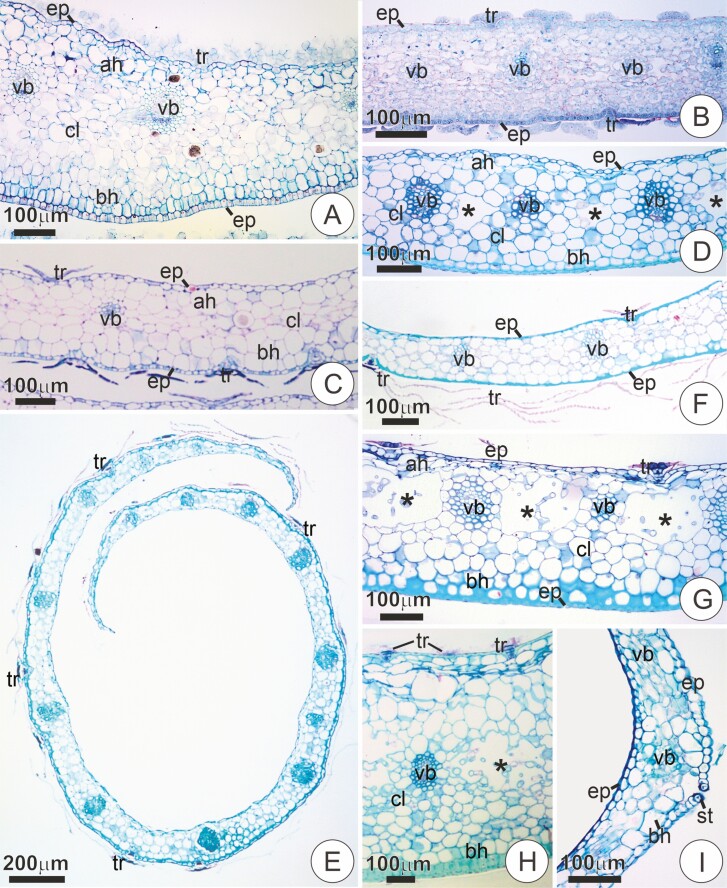
Trichome position and structural aspects of bracts in cross-sections stained in Toluidine Blue O and counterstained with Ruthenium Red. (**A**) Young bract of *Vriesea aff. bituminosa* with trichomes on the adaxial surface. (**B and C**) Young bracts of *W. viridiflora* and *V. poenulata*, respectively, with trichome coverage on both sides of the bracts. In C, note the trichomes predominant on the abaxial surface. (**D**) Mature bract of *Tillandsia tenuifolia* with glabrous epidermis. (**E**) Mature bract of *Tillandsia streptophylla* with scales predominantly in the abaxial surface. (**F**) Mature bract of *T. loliaceae* with trichomes exclusively on the abaxial surface. (**G and H**) Mature bracts of *Guzmania sprucei* and *W. cyanea*, respectively, with trichomes predominantly in the adaxial surface. (**I**) Mature bract of *B. reducta*. Notice the absence of trichomes, rarely seen in the species. Ah,  adaxial surface hypodermis; ab, abaxial surface hypodermis; cl, chlorenchyma; ep, epidermis; st, stomata; tr, trichome; vb, vascular bundle.

Peltate trichomes were often seen on the bract surface of the studied species, with position and structure varying between and within groups (Table 1). In members of the tribe Vrieseeae, the trichomes were usually present in the adaxial surface of the floral bracts (Fig. 2A; Table 1). On the abaxial surface, trichomes were only seen in some species, usually sparsely distributed. However, *Vriesea poenulata* and *W. viridiflora* displayed trichomes on both surfaces (Fig. 2B and C; Table 1).

Within species of Tillandsieae, trichomes were absent in the floral bracts of *T. araujei*, *T. stricta* and *T. tenuifolia* (Fig. 2D; Table 1). *Tillandsia loliaceae* and *T. recurvata* showed trichomes exclusively on the abaxial side (Fig. 2E; Table 1), while *T. streptophylla* and *R. crispa* had trichomes in both surfaces of the bracts, but predominantly in the abaxial one (Fig. 2F; Table 1). *Guzmania* species and *W. cyanea* comprised exceptions, their trichomes appearing mainly in the adaxial side of the bracts (Fig. 2G and H; Table 1).

The trichomes in *C. morreniana* were present in both the adaxial and abaxial surfaces of the bract. Still, they were more numerous in the adaxial one, as in most Vrieseeae species (Table 1). In *B. reducta*, trichomes were rarely seen either in the adaxial or abaxial surface of bracts (Fig. 2I; Table 1).

### Trichome structure and secretory activity

**Figure 3.**
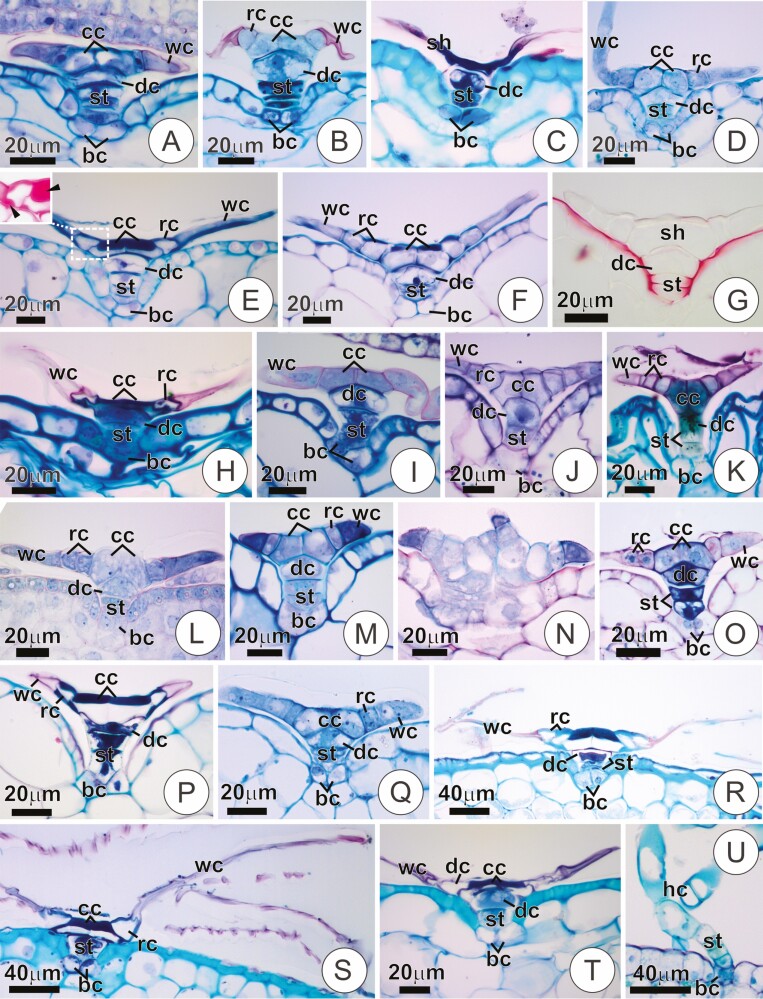
Longitudinal sections of trichome in bracts of the studied species. (**A and B**) Trichome of the adaxial surface in a young and mature bract of *Vriesea friburguensis*, respectively. Note wing cells with dense protoplast and pectic cell walls in A (in pink, as evidenced by ruthenium red), and the same cells collapsed and with empty protoplast in B. (**C**) *Vriesea erytrodactylon*. Trichome of a mature bract with a collapsed shield. (**D**) *Vriesea aff. rubra*. Trichome of a young bract displaying cells with dense protoplast. (**E and G**) *Vriesea poenulata*. Trichomes of the abaxial (E) and adaxial (F) surface of a young bract. Notice the cell wall thickenings in the central and wing cells, especially prominent in E. The insert shows a detail of the dotted area, stained with ruthenium red. In G, detail of the stalk, evidencing the cuticle (in red, Sudan Red 7B). Notice the protrusions of the cuticle towards the periclinal walls of the stalk. (**H**) Mature trichome in *Mezobromelia pleiosticha.* Note empty, collapsed wing cells with pectic walls. (**I**) Trichome of the adaxial surface in a young bract of *Goudea crysostachys* showing shield cells with dense protoplast with conspicuous nuclei. (**J and K**) *Stigmatodon goniorachis*. Trichome in a young and mature bract, respectively. In K, notice the presence of phenolics in the stalk (in green, toluidine blue). (**L**) *Werauhia viridiflora*. Young trichome on the adaxial surface with dense wing cells. (**M and N**) *Alcantarea extensa*. Trichomes of a young bract exhibiting short wing cells with dense protoplast. In N, trichomes appearing juxtaposed to one another. (**O and P**) *C. morreniana*. Trichomes of young and mature bracts, respectively. Note dense shield cells in O, and conspicuous thickening of the cell walls in P. (**Q**) Young trichome in *Guzmania roezlii*. (**R**) Trichome of the abaxial surface in a mature bract of *T. loliaceae*. Notice the thickening of cell walls in the shield portion. (**S and T**) *Tillandsia streptophylla*. Trichomes of the abaxial (S) and adaxial (T) surface of a young bract. Notice the broad wings and the cell wall thickenings in S. (**U**) Trichome in the abaxial surface of a mature bract in *B. reducta*. The head of the trichome (hc) does not differentiate in a distinct flattened shield. bc, basal cells; cc, central cells; dc, dome cell; hc, head cells; rc, ring cell; sh, shield; st, stalk; wc, wing cells.

**Figure 4.**
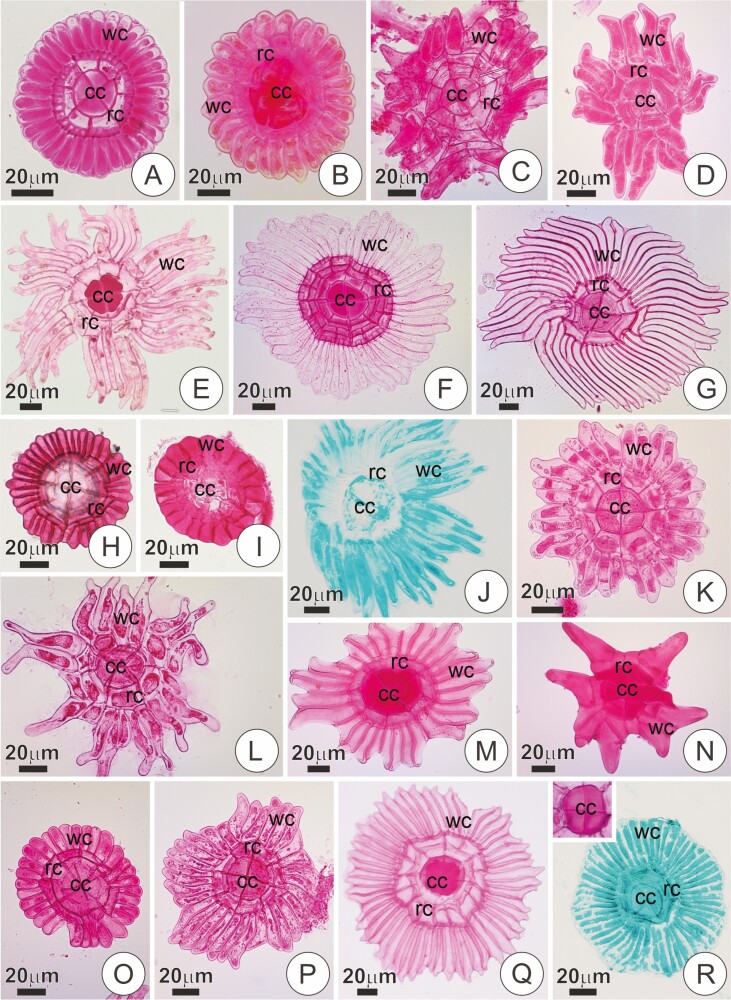
Structure of the trichome shield (surface view) in some species of Tillandsioideae stained in Ruthenium Red and Alcian Blue (J, R). (**A and B**) Trichome shield in young bracts of *Vriesea carinata* and *S. goniorachis*, respectively. Notice the regular, circular outline of the shield. (**C and D**) Trichomes of *Vriesea stricta* and *Vriesea* aff. *rubra*, respectively, with shields of irregular outline, incomplete rings and wings. (**E–G**) Trichomes of *V. poenulata*, *Vriesea psitaccina* and *Vriesea erytrodactylon*, respectively, presenting a shield with many long and narrow wing cells with free acute tips. Note the thickening of the walls in the central portion (strongly in pink). (**H and I**) Trichomes in *A. extensa* and *Alcantarea pataxoana*, respectively, with short wings of regular, circular outline. (**J**) Trichome shield in *Alcantarea farneyi* with distinct shield comprising long, narrow wing cells. (**K and L**) Trichome shields in *Goudea ospinae* and *Goudea crysostachys*, respectively. (**M and N**) Intraspecific variation in the shield structure of trichomes in *R. crispa*. Note the central portion with thick cell walls (strongly in pink). (**O and P**) Trichomes in *Guzmania pathula*, also with intraspecific variation in shield structure. (**Q**) Trichome of the adaxial surface in *Tillandsia streptophylla*. Note the shield with thickening of central cells walls and wing comprising narrow cells with free acute tips. (**R**) Shield of trichome in *W. cyanea* with regular, circular outline. The insert shows central cells with thick outer cell walls. cc, central cells; rc, ring cells; wc, wing cells.

The trichomes in all studied species were generally formed by two basal cells, a short uniseriate stalk (2–4 cells) often exhibiting a larger, dome-shaped uppermost cell (i.e. dome cell), and a distal flattened shield (Fig. 3A–T). The shield comprises a group of a few central cells surrounded by one or more rings of concentric cells and a peripheral wing formed by long, radially arranged cells (Fig. 4). In all studied species, a conspicuous cuticle was seen in the anticlinal walls of the stalk, usually forming projections towards the periclinal plane (Fig. 3G). In *B. reducta*, the trichomes did not present a recognizable flat shield. Instead, the head of the trichomes in this species comprised one or more long, free multicellular appendages (Fig. 3U).

Variation in the structure of the trichomes was observed, especially regarding the shield organization. Within tribe Vrieseeae, most *Vriesea* species, as well as *S. goniorachis*, showed trichomes with relatively well-organized, symmetrical shields in which wing cells were juxtaposed and roughly the same size, ending in a round tip that rendered a circular outline to the trichomes (Fig. 4A and B). *Vriesea* aff. *bituminosa*, *V.* aff. *rubra* and *V. stricta* were exceptional cases, showing remarkably irregular shields formed by many oblique divisions and numerous incomplete rings and wings (Fig. 4C and D). *Vriesea brusquensis*, *V. erythrodactylon*, *V. flammea*, *V. poenulata* and *V. psittacina* also showed distinct shield configurations, with numerous, narrow wing cells with variable sizes and acute free tips (Fig. 4E–G). In these species, thickening of the outer periclinal wall of central cells was usually observed (Figs 3E and F and 4E and F). In *V. poenulata*, these features are especially accentuated, the trichomes often resembling those of *Tillandsia* species (see below). In *Alcantarea* spp., trichomes were numerous, often juxtaposed in groups of two or more (Fig. 3N). The shield of the trichomes was also relatively regular, symmetrical and with wing cells juxtaposed in a circular outline (Fig. 4H and I). In these species, wing cells were remarkably short (Figs 3M and N and 4H and I), except for *A. farneyi*, in which the often-asymmetrical wing comprised long, numerous cells with variable sizes (Fig. 4J). In the studied species of *Goudea*, *G. ospinae* showed trichomes with relatively well-organized, symmetrical shields (Fig. 4K), whereas *Go. chrysostachys* displayed shields with irregular outlines in which the wing was often incomplete, and wing cells were not juxtaposed thoroughly (Fig. 4L).

Scales in *Guzmania* and *Racinaea* were usually asymmetrical with an irregular outline, but strong variation was observed (Fig. 4M–P). Trichomes in the studied *Tillandsia* species displayed shields with broad wings comprised of numerous long and narrow cells with free, acute tips (Figs 3R and 4Q). In these species, distinctive cell wall thickening was observed in the central cells, ring and wing portions (Fig. 3R–T). Similar wall features were observed in mature scales of *R. crispa* and *C. morreniana* (Fig. 3P). *W. cyanea*, in turn, showed shields similar to most *Vriesea* species. However, a conspicuous thickening was observed in the shield of mature trichomes, and the wing was also formed by numerous narrow wing cells (Fig. 4R).

In species with scales on both surfaces of the bract, we did not detect differences between the adaxial and abaxial indumentum, except for *V. poenulata* and *T. streptophylla*, in which the trichomes of the abaxial surface often showed broader shields with stronger cell wall thickening when compared with trichomes of the adaxial side, often including irregular thickening in cells of the ring (Fig. 3E, F, S and T).

**Figure 5.**
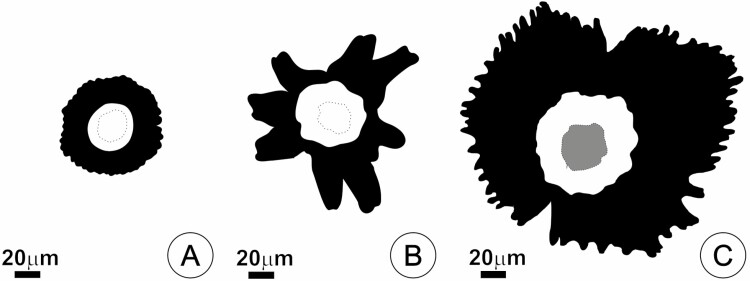
Types of trichomes in the studied species based on shield morphology. (**A**) Type A, rounded-tip wing cells thoroughly juxtaposed and forming a circular outline. (**B**) Type B, wing cells not thoroughly juxtaposed, often with incomplete wings, oblique divisions, and strongly irregular outlines. (**C**) Type C, many celled-wing formed by narrow cells with free acute tips, often with thickening of the walls, especially those of the central portion. Black area = wing, White area = central portion, the dotted line indicates the central cells position. The grey area represents thickening of the outer periclinal walls.

Based on the variable structure of the shield, trichomes within the studied Tillandsioideae species can be roughly divided into three types: type A, with rounded-tip wings cells thoroughly juxtaposed and forming a circular outline (Fig. 5A); type B, with wing cells not thoroughly juxtaposed, often with incomplete wings, oblique divisions and irregular outline (Fig. 5B) and type C, presenting broad, many-celled wing formed by narrow cells with free acute tips, frequently with characteristic thickening of the cells at maturity, especially those of the central portion (Fig. 5C). Table 2 list the main features of each trichome type.

**Table 2.** Characterization of trichome types based on shield arrangement.

Trichome type	Features
A	• Shields with circular outline • Wing cells with rounded-tips • Wing cells thoroughly juxtaposed
B	• Shields with irregular outline • Rings and wings often incomplete • Oblique cellular divisions • Wing cells not thoroughly juxtaposed
C	• Wing relatively broad, many-celled • Wing cells narrow, with free acute tips • Cell walls of shield irregularly thickened

**Figure 6.**
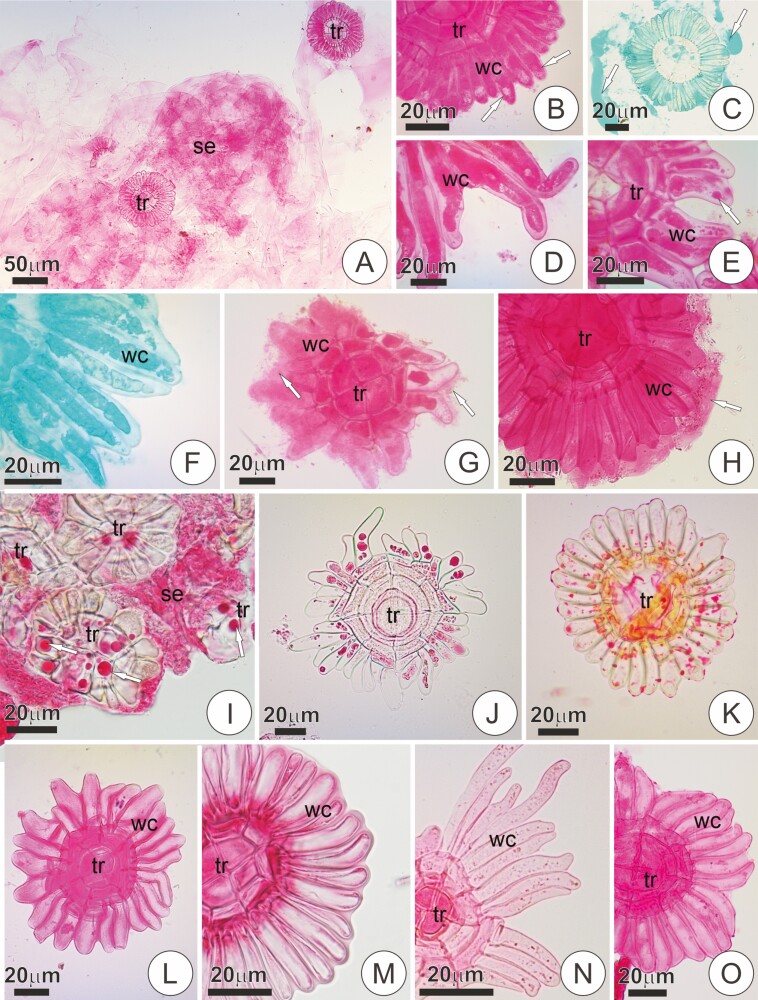
Secretions associated with trichomes in some Tillandsioideae species stained with Ruthenium Red, Alcian Blue (C and F) and Sudan Red 7B (I–K). (**A**) *V. carinata*. Film of secretion stained positive for mucilage (in pink). (**B**) *Vriesea friburguensis*. Portion of a trichome shield showing wing cells with dense protoplast filled with mucilage (arrows). (**C**) Trichome shield of *Vriesea paraibica* associated with mucilage (arrows). (**D and E**) Portions of the shield in *Goudea chrysostachys* and *Guzmania ronhofiana*, respectively. Note wing cells with dense protoplast filled with mucilage (in pink). (**F**) *Werauhia viridiflora*. The protoplasts of wing cells are filled with mucilaginous content (in blue). (**G and H**) Shield of trichomes in *C. morrenniana* and *Tillandsia streptophylla*, respectively, associated with a film of mucilage (arrows). (**I–K**) Trichomes in *A. pataxoana*, *Vriesea stricta* and *S. goniorachis*, respectively, associated with lipid secretion (in red). Secretion is seen as lipid droplets inside (I and J) or over (K) the shield cells, and as a film of secretion around the shield (I). (**L–O**) Trichome shield from mature bracts of *R. crispa*, *Vriesea carinata*, *W. viridiflora* and *Guzmania pathula*, respectively. Notice the empty cell lumens in the shield, especially in the wing cells. se, secretion; tr, trichome; wc, wing cells.

Trichomes were associated with secretion in several species (Table 1), either as a film around and over the shields or accumulated inside the protoplast in trichomes of young bracts (Fig. 6A–K). The secretion in *V.* aff. *bituminosa* and *V. fenestralis*, appeared as a strongly heterogeneous material (not shown). Secretions tested positive for mucilage in all cases (Fig. 6A–H; Table 1), whereas lipids were observed exclusively in *A. burle-marxii, A. extensa*, *A. pataxoana*, *S. goniorachis, V.* aff. *bituminosa, V. botafogensis, V. fenestralis*, *V. minarum*, *V. platynema* var. *rosea* and *V. stricta* (Fig. 6I–K, Table 1). In *C. morreniana* and *T. streptophylla*, the shield of the adaxial trichomes was often seen in association with a film of mucilaginous secretion (Fig. 6G and H), even though no secretion was observed to accumulate on the surface of the bracts. In the latter and in *V. poenulata*, where trichomes were distinct between abaxial and adaxial sides, secretion was only observed in the trichomes of the adaxial portion.

Differences in the structure of trichomes from young and mature bracts were evidenced by the presence of living or dead shield cells, cell wall structure and phenolic deposition. In scales of young trichomes, cells of the shield showed living protoplasts and thin pecto-cellulosic walls; cells of the wing often showed distinctive pectic walls and dense protoplast with conspicuous nuclei (Fig. 6A–H, see also Fig. 3). In turn, the shield of trichomes in mature bracts usually undergone increased vacuolation and protoplast loss, showing empty lumens (Fig. 6L–O, see also Fig. 3). In this stage, the collapse of shield cells was common. This collapse was either exclusively of the wing portion (Fig. 3B) or the entire shield (Fig. 3C). Deposition of phenolics was also observed in mature trichomes, mainly in the stalk cells (Fig. 3K). Trichomes in species of *Tillandsia* and *V. poenulata* did not show differences between the young and mature portions. Trichomes appeared fully expanded in these species, even in the samples of young bracts.

### Bract anatomy

**Figure 7.**
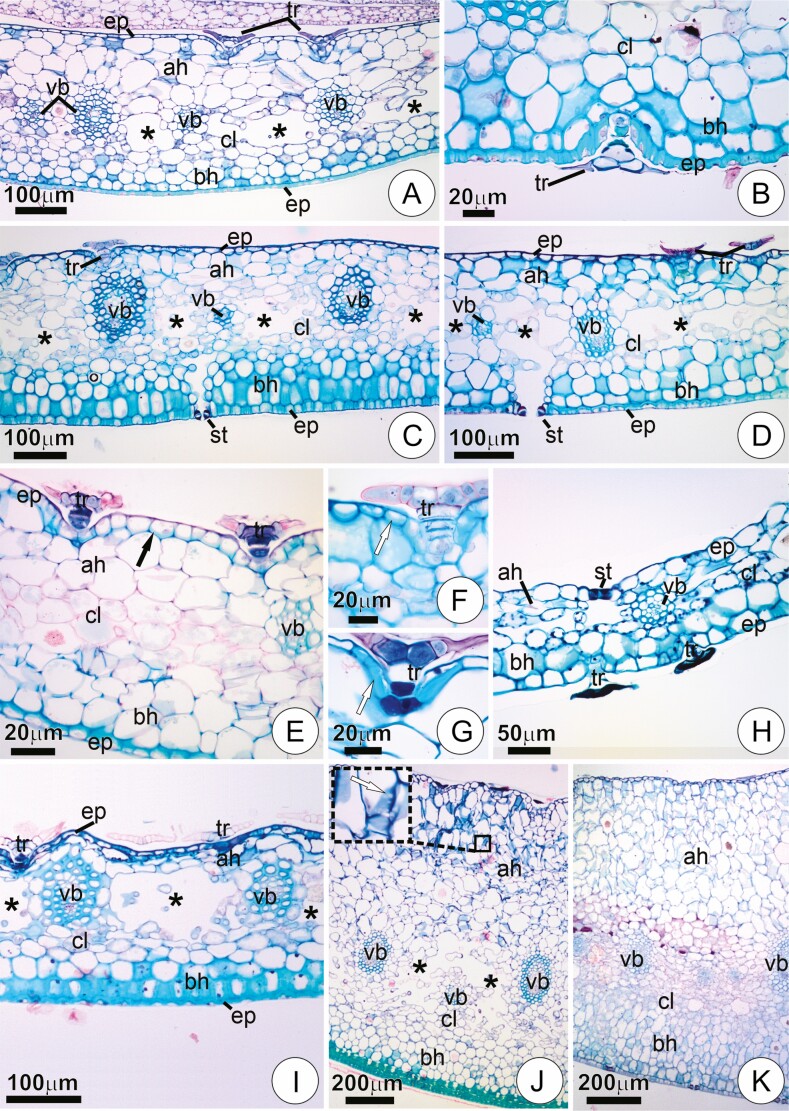
Transversal sections showing bract anatomy in the studied species, stained with Toluidine Blue O and counterstained with Ruthenium Red. (**A and B**) *Vriesea carinata*. Overall structure of mature bracts. In B, detail of the abaxial portion, with a trichome. Note the sclerified epidermis and abaxial hypodermis with isodiametric cells and little distinction from the adjacent chlorenchyma. (**C and D**) Mature bracts of *Vriesea ruschii* and *Alcantarea compacta*, respectively. Notice stomata in the abaxial surface with substomatal chamber contacting the air-lacunae (asterisks). (**E**) Mature bract of *C. morrenniana* with sclerification in the epidermis at adaxial (arrow) and abaxial surface, and hypodermis with large cells forming a water-storage tissue, including in the abaxial side. (**F and G**) Mature trichomes of *Goudea ospinae* and *Guzmania sprucei*, respectively, with localized sclerification around the trichome stalk (arrows) of the adaxial bract surface. (**H**) Mature bract of *Racineae crispa*. Note trichomes of the abaxial surface, and stomata in the adaxial one. (**I**) Mature bract of *Goudea crysostachys.* Note the abaxial surface with heavily sclerified epidermis and hypodermis, as well as pronounced air-lacunae (asterisks). (**J and K**) Bracts of *Alcantarea extensa* and *S. goniorachis*, respectively, with conspicuous water-storage hypodermis towards the adaxial side. The insert in J shows detail of cells of the water-storage parenchyma with thin walls with numerous primary pit fields (arrow). ah, adaxial hypodermis; bh, abaxial hypodermis; cl, chlorenchyma; ep, epidermis; st, stomata; tr, trichome; vb, vascular bundle.

The floral bracts of the studied species comprised a uniseriate epidermis, a heterogenous mesophyll, and a vascular system consisting of collateral vascular bundles (Fig. 7A–K). The epidermis had small cells, usually with thick anticlinal and periclinal walls in the abaxial portion forming a sclerotic layer (Fig. 7B). Sclerification of the entire epidermis on the adaxial surface was uncommon, occurring only in *C. morreniana*, *Go. chrysostachys, T. streptophylla, V.* aff*. rubra* and *W. cyanea* (Fig. 7E). Localized sclerification of the adaxial surface occurred around the basal cells of adaxial scales in *G. ospinae* and *G. sprucei* (Fig. 7F and G). Except for *R. crispa*, stomata were presented exclusively on the abaxial surface of the bracts (Fig. 7C and D). In *R. crispa*, however, stomata were observed on both surfaces of the bract, predominantly on the adaxial side (Fig. 7H).

The mesophyll in all species was heterogeneous and usually comprised a central chlorenchymatous tissue loosely limited by a multilayered hypodermis placed on both adaxial and abaxial surfaces (Fig. 7A, C and D). On the adaxial surface, the hypodermis is usually a water-storage parenchyma comprised of large cells with thin walls perforated by numerous primary pit fields (Fig. 7A and J). The thickness of this water-storage tissue was usually higher towards the midrib region; it varied considered between species, being especially prominent in *A. burle-marxii, A. extensa, A. pataxoana* and *S. goniorachis* (Fig. 7J and K). This tissue was usually comprised of one to two layers of cells in *T. loliacea, T. mallemontii*, *T. recurvata* and *T. tenuifolia* (e.g. Fig. 2D and I).

The abaxial hypodermal cells were either isodiametric or anticlinally elongated, and their delimitation from the inner tissue was not always clear (Fig. 7B and C). Distinct degrees of sclerification were seen in hypodermis at the abaxial side, sometimes only with discrete thickening of the outer layers (Fig. 7A, B and D). Alternatively, sclerified tissue was observed in up to three layers subtending the epidermis at the abaxial side (Fig. 7C and I). In *A. compacta, C. morreniana*, *R. crispa, T. streptophylla* and *V. poenulata*, the abaxial surface showed a hypodermis with large cells of thin walls with numerous primary pit fields that seem to comprise a water-storage tissue (Fig. 2C, Fig. 7D and E). The same situation was noted in *B. reducta*, although in this species a water-storage tissue was not present towards the adaxial side, being limited to the abaxial one (Fig. 2I). The chlorenchyma was usually restricted to a central spongy portion, often not well delimited (Fig. 7A–E, H and I). Intercostal air lacunae developed within the chlorenchyma in most species, being absent only in *C. morreniana, B. reducta, R. crispa*, *T. loliacea*, *T. mallemontii* and *T. recurvata (*Figs 2F and I and 7H). In the remaining species, air channels were always observed, ranging from poorly developed, as in *G. wittimackii, S. goniorachis, T. araujei* and *T. tenuifolia* (Figs 2D and 7K), to rather broad and conspicuous, as in mature bracts of *Goudea* species, *Guzmania patula*, *G. roezlii*, *G. sprucei, M. pleiosticha, V.* aff. *rubra* and *W. viridiflora* (e.g. Fig. 7I). In *V.* aff. *bituminosa*, the entire chlorenchyma was formed by strongly spongy parenchyma in which air channels proper were not observed (Fig. 2A). An assimilating tissue was clearly distinct only in *A. farneyi*, *C. morreniana*, *M. pleiosticha, T. streptophylla, V.* aff. *rubra, V. flammea, V. lubersii, V. poenulata, V. scalaris* and *W. viridiflora* in which conspicuous chloroplasts could be seen (e.g. Fig. 7E).

**Figure 8.**
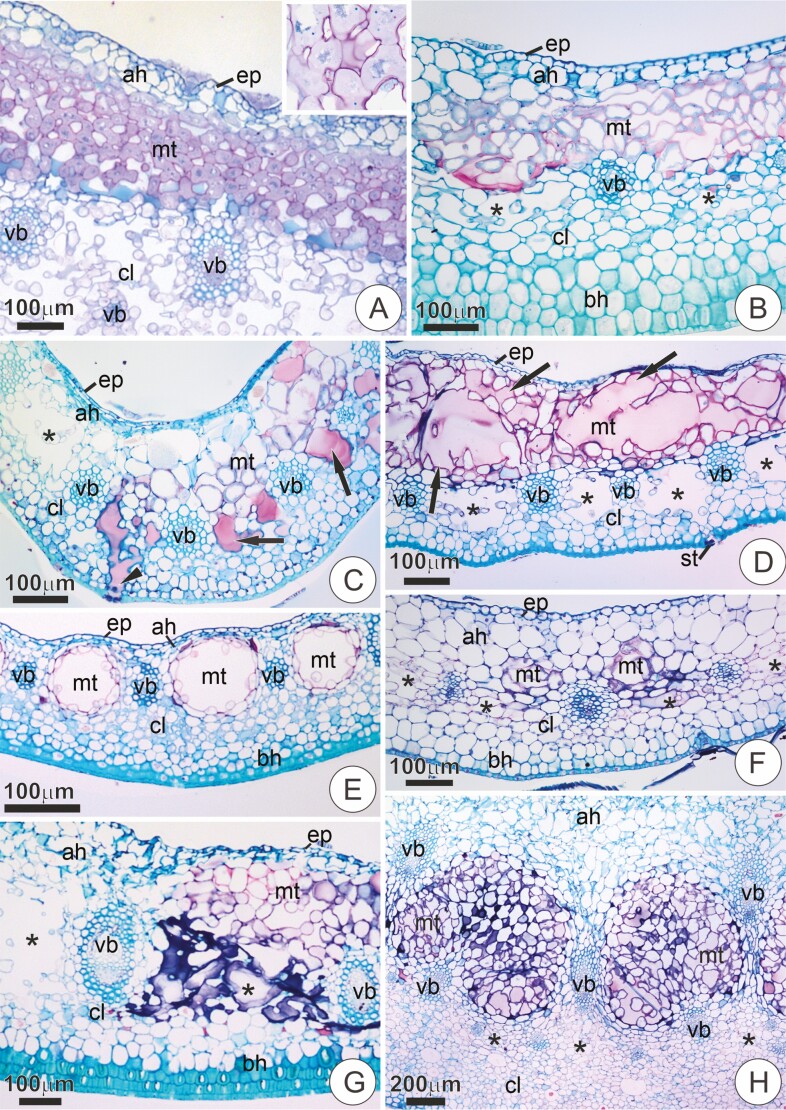
Transversal sections of the midrib portion in bracts of some Tillandsioideae species stained in Toluidine Blue O and counterstained with Ruthenium Red. (**A**) *Vriesea aff bituminosa*. Notice the mucilaginous tissue between the hypodermis of adaxial surface and the inner portion of the chlorenchyma. The insert shows a detail of the mucilaginous tissue evidencing cells with dense protoplast and large intercellular spaces. (**B**) *Vriesea botafoguensis*. (**C**) *Vriesea brusquensis*. Notice the air-lacunae filled with mucilage (arrows) extending to the substomatal chamber (arrowhead). (**D**) *Vriesea erytrodactylon*. The mucilaginous tissue takes over the entire water parenchyma, making direct contact with the epidermis of the adaxial surface. Notice the large intercellular spaces filled with mucilage (arrows). (**E**) *Mezombromelia pleiosticha*. The mucilaginous tissue exhibits well-defined lumens between the vascular bundles. (**F**) *Vriesea poenulata*. Notice the poorly developed mucilaginous tissue appearing as two small circular lumens in the central portion. (**G**) *Alcantarea burle-marxii*. Region of transition between the margin (left) and midrib portion (right) of a mature bract. Notice the mucilaginous tissue differentiated within the hypodermis of the adaxial surface and the subtending air channel (asterisks) filled with mucilage. (**H**) *Stigmatodon goniorachis.* Notice the mucilaginous tissue along the vascular bundles, but not reaching the small air-lacunae below (asterisks). ah, adaxial hypodermis; ab, abaxial hypodermis; cl, chlorenchyma; ep, epidermis; mt, mucilaginous tissue; v, vascular bundle; asterisks, air-lacunae.

Within Vrieseeae, a distinct tissue characterized by the presence of protoplast-dense cells with pectic walls and large intercellular spaces filled with mucilaginous content was present in the midrib region of the bracts (Table 1, Fig. 8A–H). This tissue was usually conspicuous, developing between the adaxial hypodermis and the chlorenchyma (Fig. 8A–H). In some cases, this tissue appeared as well-defined channels (e.g. *M. pleiosticha*, *V. poenulata*; Fig. 8E and F), but in several species, there was no clear delimitation of a lumen (Fig. 8A–C, G and H). The position of this tissue in relation to the vascular system varied in two ways: appearing either above the vascular bundles (Fig. 8A, B and D) or intercalating with the veins in an intrusive manner (Fig. 8C, F and G). In the latter, the large intercellular spaces of this tissue merge with the air-lacunae, making it difficult to distinguish from the subtending chlorenchyma (Fig. 8C, F and G). In fact, in several species, mucilage was observed filling the air-lacunae (Fig. 8C, F and G), sometimes extending over to the substomatal chambers (Fig. 8C). In *S. goniorachis*, this tissue appeared between the vascular bundles but did not seem to touch the poorly developed air-lacunae (Fig. 8H).

Moreover, in some sections of the studied bracts, this ‘mucilaginous’ tissue appeared to substitute the adaxial hypodermis completely, directly contacting the epidermis towards the adaxial surface (e.g. *V. erythrodactylon*, *V. ruschii*; Fig. 8D). Conversely, in *M. pleiosticha*, this tissue seemed to substitute the air-lacunae, forming well-defined channels delimited by cells with distinct pectic walls (Fig. 8E).

The collateral vascular bundles were usually disposed in the median portion, with varying callibers, intercalating with the air lacunae. In most species, these bundles were usually surrounded by a sclerified sheath (Fig. 7C–E, I–J; Fig. 8).

## Discussion

### Secretory activity

Our results suggest that a secretory activity associated with floral bracts is not uncommon among tillandsioid bromeliads (present in 44 out of 52 sampled species). The presence of dense cells and conspicuous nuclei, as observed in young trichomes of the adaxial bract surface, indicates their secretory function (Fahn 1979, 2000). The observations of secretion (either as films or inside the protoplast) and results from histochemical tests for mucilage and lipids further corroborate the secretory activity of these scales.

The remarkable distinctions observed between trichomes of young and mature bracts (e.g. shield cells with dense protoplast and conspicuous nuclei vs. empty lumens and collapsed cell walls at maturity) indicate that the secretory activity is limited to young trichomes in bracts not fully expanded (see Ballego-Campos and Paiva 2018; Ballego-Campos *et al.* 2020). In the studied species of *Tillandsia* and *V. poenulata*, the absence of young developing trichomes is explained by a particularly early and rapid development.

The distinct aspect of the trichome wing cells in several of the studied species, mainly the relatively denser protoplast and distinctive pectic walls, suggest that this region constitutes a separate functional portion of the trichome, which is especially involved in secretion production and release, as suggested previously in *W. cyanea* and *A. blanchetiana* (Ballego-Campos and Paiva 2018; Ballego-Campos *et al.* 2020). However, secretory activity in the central portion of the trichomes cannot be excluded without an in-depth analysis since the protoplast density and cell wall structure might be difficult to distinguish in some cases. For instance, in very young trichomes, the wing and the central portion might present active protoplasts related to cell expansion and differentiation. Likewise, dense cytoplasm in the axial portion of bromeliad scales might be associated with water-absorption control (Brighigna *et al.* 1988; Ballego-Campos *et al*. 2020). Furthermore, due to the often-irregular development of trichomes in some species, including incomplete rows of cells and oblique divisions, wing and ring cells cannot always be accurately determined regarding their developmental origin.

### Trichome structure and position

Trichomes of type A seem to be characteristic of Vrieseinae species, although also occurring in *G. ospinae* (Cipuropsidinae), and *W. cyanea* (Tillandsieae) (Table 1). We believe this type A trichome might be a synapomorphic trait of subtribe Vrieseinae, arising independently in *W. cyanea* and *G. ospinae*. Inflorescences of *Wallisia* species are remarkably similar to the flat, lanceolate and distichous inflorescences of many *Vriesea* species. Likewise, *G. ospinae* also shows flat, strongly complanate distichous spikes (see Smith and Downs 1977; Luther 1983; Barfuss *et al*. 2016). Furthermore, the presence of thick cell walls in the central portion of *W. cyanea*, as well as numerous wing cells, indicate that these scales do not fully correspond structurally with Type A trichomes (i.e. non-homologous). *V.* aff. *bituminosa* and *V. stricta* presented scales more closely related to type B trichomes (e.g. irregular, asymmetrical shiels), possibly representing a transformation in this character (Table 1).

Type B trichomes seem to be particularly associated with members of the tribe Cipuropsidinae (Table 1), but the significance of this feature, either from an evolutive or ecological perspective, needs further investigation. The consideration of type B trichomes as an informative trait is particularly challenging due to the lack of representants of some genera in our analysis (*Jagrantia, Josemania*, *Lutheria*, *Zizkaea*) and the unresolved phylogeny of the *Cipuropsis-Mezobromelia* Complex (Barfuss *et al.* 2016; Machado *et al*. 2020). The presence of a strongly irregular head in the inflorescence trichomes of the outgroup species *B. reducta* might suggest that an irregular distal portion is plesiomorphic in the family.

Type C trichomes are similar to foliar scales of most *Tillandsia* species, particularly those of extreme ‘atmospheric’ lifeforms, which also show broad, many-celled wings and distinct cell wall thickening (Tomlinson 1969; Benzing 1976, Table 1). Regarding these trichomes, such features are often interpreted as adaptations that ensure rapid and efficient water absorption via a sophisticated pump-like mechanism (Benzing 1976). In fact, the scales in floral bracts of the extreme epiphytes *T. loliacea* and *T. recurvata* are very similar to the complex and sophisticated structure of leaf-absorbing trichomes in atmospheric tillandsias. Nonetheless, trichomes with type C features also appear in other genera (see Table 1).

Outside *Tillandsia*, type C features seem to be related to smaller, xeric and tankless lifeforms in at least three cases: in *V*. *poenulata*, *A*. *farney*i and *R. crispa*. *Vriesea poenulata* is a small species whose narrow linear-triangular leaf blades form a utriculiform rosette rather than an infundibuliform one (Gomes-da-Silva and Costa 2011). Furthermore, an ­analysis of morphological and anatomical data (not including inflorescence trichomes) showed that this species shares similarities with *Tillandsia* species, including extreme ‘atmospheric’ taxa (e.g. *T. stricta*, *T. tenuifolia*; Gomes-da Silva *et al.* 2012). Similarly, the distinctly broad wings in trichomes of *A. farneyi* might also reflect adaptations to a reduced, tankless lifeform. Unlike all other *Alcantarea* species studied, this species has a graminoid habit, with narrow leaf blades forming a tankless sub-bulbous rosette that stores little to no water (Versieux 2009). *Racinaea crispa* is also a small epiphyte with narrowly triangular blades (Smith and Downs 1977), unlikely to rely on stored water in the rosette. Thus, as generally considered for the vegetative shoot, we believe that trichomes in the floral bracts are also specialized in xeric species that cannot rely on roots or well-developed phytotelmata for water and nutrient supply (see Benzing 1976, 2000; Givnish 2014).

However, it is important to note that these assumptions are based on the premise that inflorescence trichomes, like foliar scales, can absorb water. There is no empirical data on water absorption by inflorescence trichomes of bromeliads, and efforts in this direction are strongly encouraged. Nonetheless, the absorptive capacities of inflorescence trichomes seem to be supported at least by their structure, namely the similar arrangement of cells in comparison with foliar scales, the metabolic active cells in the axial portion, and perhaps more importantly, the presence of an identical cuticular pattern which, in turn, is considered fundamental to the one-way valve-like mechanism of the absorptive foliar scales (Tomlinson 1969; Benzing 1976, 2000; Brighigna *et al.* 1988; Raux *et al.* 2020).

Regarding the position of the trichomes, our results show that trichomes of the adaxial surface are strongly related to secretory function. In contrast, the indumentum in the underside of bracts is either scarce (as in most *Vriesea* and *Alcantarea* species) or present type C features (e.g. *T. loliacea*, *T. streptophylla*, *V. poenulata*). This differential distribution of trichomes suggests that distinctive selective pressures have acted on the adaxial and abaxial surfaces of floral bracts in Tillandsioideae species. At least in tribe Vrieseeae, and in part of the Tillandsieae (i.e. *Guzmania* and *Wallisia*), these pressures seem to have favoured a glandular indumentum in the adaxial surface of floral bracts, perhaps associated with a more mesic, tank-forming habit. Within the remaining Tillandsieae, our sampling does not allow any meaningful insight regarding possible trends or patterns. The presence of secretion in *T. streptophylla* and its absence in the other sampled tillandsias is unclear. It is likely that the lack of glandular trichomes in the underside of most atmospheric species is related to a reduction in the size of the inflorescence (and of the overall plant body) allied to additional means of water acquisition and conservation (e.g. specialized leaf-absorbing trichomes, Crassulacean acid metabolism [CAM] photosynthesis); see Benzing 2000; Givnish *et al.* 2011, 2014). *Tillandsia streptophylla* possess considerably larger and branched inflorescences in which the flowering period extends to a much broader time span. Nonetheless, future efforts with a broader sampling of *Tillandsia* are needed to clarify these observations further.

The presence of trichomes in both *W. viridiflora* bract surfaces is unique because it is the only species in which a conspicuous indumentum in the underside was not associated with type C trichome features. As previously stated, within Vrieseeae, trichomes on abaxial surface were conspicuous only in *V. poenulata*, which differs dramatically in size and habit. It is unclear whether this feature might be informative, either as a diagnostic character or in an evolutive or ecological perspective. Perhaps significantly, we noted that the floral bracts in *W. viridiflora* tend to become paleaceous shortly after their expansion, possibly as part of a senescence process.

### Aspects of the secretions and their ecological function

Histochemical tests revealed that the exudate produced by floral bracts in the studied species might be exclusively mucilaginous or a mixed secretion comprised of mucilage and lipids. Mucilaginous and mixed secretions are common products in secretory systems associated with the young reproductive and vegetative axis of several plants and are usually involved in protective roles (Fahn 1979; Thomas 1991; Tresmondi *et al*. 2015). In this sense, the secretory trichomes in the adaxial surface of floral bracts likely act as colleters, producing an exudate that covers the young portions and avoid desiccation, herbivores or pathogens (Thomas 1991; Paiva 2012; Mayer *et al.* 2013; Tresmondi *et al.* 2015; Paiva *et al*. 2021; Gonçalves *et al.* 2022; see also Ballego-Campos *et al*. 2023).

The lipid secretion in floral bracts of *Alcantarea* species and *S. goniorachis* does not support the proposition that lipophilic exudates are exclusive of certain members of the traditional *Vriesea* sect. Xiphion. However, the apparent association of lipid secretion with species that often grow in rocky, exposed habitats is remarkable; it might suggest a convergence related to the heliophytic habit (see Ballego-Campos *et al*. 2023). Lipophilic secretions can protect young and developing organs from irradiance, excessive transpiration and heat by increasing reflectance and resistance to water loss (Dell 1977; Machado *et al.* 2012; Tresmondi *et al.* 2015).

The distinct secretory activity observed in *C. morreniana* and *T. streptophylla* (e.g. localized exudation of mucilage, limited to the trichome shield) cannot be interpreted functionally in the same manner as the secretion in the remaining species. An inconspicuous coverage of secretion is likely not effective in the protective roles discussed above. Instead, the mucilage secretion observed in *C. morreniana* and *T. streptophylla* might be exclusively related to the putative absorptive capacity of the trichomes in these species, providing a highly hygroscopic surface associated with the scales.

Regarding the aspect and abundance of the secretion in the studied species, exudates with sticky, greasy and oily aspects are usually related to the presence of considerable amounts of lipophilic compounds, such as lipids or even terpenes (e.g. *V. fenestralis, V. stricta*; see Ballego-Campos *et al*. 2023). Such exudates behave more like resins or gums, assuming a solid, vitreous aspect when exposed; in contrast, polysaccharide-rich secretions appear as gelatinous exudates that decrease dramatically in volume upon exposure, often reducing to thin films of colourless material. While swelling, gelification and hardening are among general characteristics of plant resins and mucilages (Langenheim 2003; Western 2012; Ballego-Campos and Paiva 2018), secretions in inflorescences of the studied species are extremely variable and do not allow for characterizations based on aspect. The wide variety of forms in which the secretion can be perceived might be explained based on the secretion composition and its exposure to environmental conditions, such as high/low humidity and precipitation. In the first case, lipophilic and hydrophilic components are often present in a heterogenous mixture, which might be secreted in distinct moments of the trichome secretory phase (Ballego-Campos *et al*. 2023.). Thus, the aspect of the secretion might depend on which class of substance is present, their ratio, and, potentially, the stage of development of a given bract/trichome. Likewise, exposure to the environment might alter the aspect of plant secretions (Cunningham *et al*. 1977; Langenheim 2003; Paiva 2009; Ballego-Campos and Paiva 2018). In mature bracts, mucilage could also be washed off or even reduced in volume during periods of drought. These observations call for careful use of secretion as a diagnostic trait. A more accurate description of the secretion, preferably based on some form of chemical scrutiny (e.g. histochemical, solubility tests), should be employed to produce a comprehensive understanding of the biology of secretion in inflorescences of bromeliads.

### Bract anatomy

Floral bracts of Tillandsioideae species seem to share several features with the overall structure of bromeliads leaves, mostly notably xeric traits such as sclerotic epidermis and the presence of hypodermis, which is often differentiated in a water-storage tissue (Tomlinson 1969; Santos-Silva *et al*. 2013; Faria *et al*. 2021). In leaves, sclerification of the adaxial epidermal surface is common (Tomlinson 1969; Santos-Silva *et al*. 2013; Faria *et al*. 2021), a feature mostly absent in the studied floral bracts. The presence of this trait in *Go. chrysostachys* comprise another distinction of this species with *G. ospinae*, which might corroborate the position of *G. chrysostachys* as more closely related to the *Cipuropsis-Mezobromelia* Complex (Machado *et al*. 2020). In fact, a sclerotic epidermis toward the adaxial surface was also observed in *V.* aff. *rubra*, another member of the *Cipuropsis-Mezobromelia* Complex. Future investigations should verify whether or not species of *Vriesea* (*lato sensu*) included in this complex share the same feature. Nonetheless, the evolution of this trait needs further scrutiny since it also appears in non-related species such as *C. morreniana*, *W. cyanea* and *T. streptophylla*.

Our results suggest that the presence of a sclerotic hypodermis might be more associated with a structural ­function (mechanical support or protection against herbivores) than a xeric trait (see also Santos-Silva *et al*. 2013; Faria *et al*. 2021). Many species that inhabit xeric environments or are usually considered highly xeromorphic did not show a mechanical hypodermis (e.g. *A. compacta*, *A. farneyi*, *T. loliacea* and *T. stricta*). Conversely, mesic species often present such characteristics (*Guzmania* species, *M. pleiosticha*, *V. warmingii*).

In *R. crispa*, the presence of stomata predominantly in the adaxial surface of the bracts is a unique feature. Further investigations of the bract anatomy in species of *Racinaea* might indicate if this is an autapomorphic trait or a shared feature and, therefore, a synapomorphy of the genus. The presence of stomata on the adaxial surface might be an adaptive trait related to a reduction of water loss via excessive transpiration. Unlike most studied species, *R. crispa* has inflated floral bracts (Smith and Downs 1977), not adpressed to the developing flower. This arrangement, while allowing for gas exchange, might increase the boundary layer in the adaxial face, thus increasing the resistance to water vapour diffusion and minimizing water loss by excessive transpiration.

The poor distinction of the chlorenchyma in most of the studied species indicates that floral bracts do not contribute significantly to carbon assimilation. In leaves, the occurrence of air-lacunae within the chlorenchyma is usually ­interpreted to comprise an internal aerating system, especially for the submerged tissues in tank-forming species (Tomlinson 1969). Their presence in bracts seems to challenge this view. Alternatively, air-lacunae might be a plesiomorphic trait of the Poales, in bromeliads possibly related to vapour-phase transport (Males 2016).

The presence of water-storage tissues is a common feature of bromeliad leaves (Tomlinson 1969; Santos-Silva *et al*. 2013; Faria *et al*. 2021) and has been shown to provide water to the chlorenchyma in conditions of drought (Stiles and Martin 1996; Nowak and Martin 1997). However, water stored in the bracts might have a distinct fate since the assimilating tissue appears inconspicuous in most species.

In the midrib portion of bracts, a mucilage-secreting tissue is a unique feature, apparently characteristic of subtribe Vrieseinae, but also appearing in *M. pleiosticha*. Records of this tissue in the literature are virtually absent. We believe this tissue is not homologous to the existing reports of mucilage in water-storage tissues of some bromeliad leaves (see Krauss 1949; Tomlinson 1969; Voltolini *et al.* 2009). Aside from being associated with the leaves, these reports clearly indicate mucilaginous content within the large, thin-celled walls of the water-storage parenchyma. In our observations, however, the mucilage-secreting tissue of the bracts is a distinct, delimited tissue that bears no resemblance with the water parenchyma itself. It has cells with dense protoplast, and large intercellular spaces filled with a mucilaginous secretion. Recently, Silva *et al.* (2020) showed the presence of secretory canals in the peduncle bracts in species of the *V. oligantha* Complex, which seem to correspond to the mucilaginous tissue observed in the present study. Nevertheless, we preferred to use the term ‘mucilaginous tissue’ in describing these structures since there is no delimitation of a secretory epithelium and, especially, a lumen in many of the observed cases. We believe these are important features in the delimitation of channels/cavities as traditionally recognized (Fahn 1979). Relatively well-delimited lumens seem to be common in sepals of *Guzmania* species (*pers. observation*, but see also Sajo *et al*. 2004) and might occur in other species of Tillandsioideae. The contents of the mucilaginous tissue do not appear to exude to the outside of the bract, as we did not note any signs of release pathways, except possibly the stomatal pore in *V. erythrodactylon*. Nevertheless, mucilage exudation through the stomata would not explain any significative contribution of these structures to the secretion observed on the adaxial side. Mucilage retention in the interior of the plant body, either inside the cells or in internal spaces, is not uncommon among plants (Fahn 1979; Gregory and Baas 1989; Barros *et al*. 2023). Internal mucilage is usually associated with water storage, protection from high irradiance, protection against herbivory (upon mechanical damage) and carbohydrate storage (Fahn 1979; Gregory and Baas 1989; Costa *et al*. 2021; Fortuna-Perez *et al*. 2021). Due to the hygroscopic capacities of mucilage, the mucilaginous tissue could function similarly to the water-storage tissue, retaining water and avoiding desiccation.

### Evolutionary and taxonomic notes

As previously stated, secretion in species of all major groups of Tillandsioideae suggests that the occurrence of secretory scales in inflorescences of this subfamily is not uncommon. In fact, we believe that this feature might comprise an ancestral trait of tillandsioid bromeliads, which then suffered reversals in *V. guttata*, *R. crispa*, and at least in some *Tillandsia* species (*T. araujei, T. loliacea, T. malemontti, T. recurvata, T. stricta, T. tenuifolia*). Particularly, the presence of non-secretory trichomes in the outgroup species *B. reducta* and the evidence of secretion by bract trichomes in the early diverging genus *Catopsis*, corroborates this view. Nevertheless, the prospection of secretion in inflorescences of this subfamily is a challenging enterprise, and a broader sampling, including additional species of *Brochinnia*, *Racinea* and *Tillandsia*, as well as members of the monotypic tribe *Glomeropitcairnieae*, is necessary to corroborate this proposition further. In *V. guttata*, the absence of secretion suggests an autapomorphic trait by reversal, at least within *Vriesea*. In *R. crispa*, besides the possible effects of size reduction previously discussed, the presence of stomata on the adaxial surface of bracts might indicate that distinctive selective pressures acted on the bracts in this species, perhaps also accounting for the lack of secretion.

The secretion observed in inflorescences of *A. blanchetiana* (Ballego-Campos and Paiva 2018) is interpreted as convergence. *Aechmea* and several other Bromelioideae taxa appear to share various convergent traits with members of Tillandsioideae, including the tank-epiphyte habit (Givnish *et al*. 2014).

Our results seem to corroborate the finding of Machado *et al*. (2020) regarding the status of *Goudea*, which these authors recovered as a non-natural group, *G. chrysostachys* are more closely related to the *Cipuropsis-Mezobromelia* Complex than *G. ospinae*. These two species differ by the configuration of the trichome shield, which presents Type A features in *G. ospinae*, and the sclerification of the adaxial surface of the epidermis (absent in *G. ospinae*). Moreover, *G. chrysostachys* share type B trichomes with *M. pleiosticha* and *V.* aff*. rubra* (*Cipuropsis-Mezobromelia* Complex) and the presence of an adaxial sclerotic layer with *V.* aff *rubra*.

We also found evidence that further corroborates the separation of *W. cyanea* from *Tillandsia* as proposed by Barfuss *et al.* (2016). This species differs from the remaining studied *Tillandsia* species by the presence of abundant secretion in the inflorescences and trichomes with type A features predominantly in the adaxial surface of the bracts.

### Directions for future research

Our results show that while the vegetative axis of bromeliads is relatively well-known, our knowledge of the anatomy, ecophysiology and evolution of the reproductive axis needs further scrutiny. Some points for future research in this area include:

(a) *broader analysis of the secretory capacity of scales in bromeliad inflorescences*. This includes broader sampling within Tillandsioideae, but also in the remaining subfamilies. The presence of secretory scales in *A. blanchetiana* (Ballego-Campos and Paiva 2018) might be an isolated instance of a much pervasive feature.
(b) *detailed accounts of the anatomy and development of the ‘mucilaginous tissue’.* A better understanding of this unusual secretory tissue is strongly encouraged. Efforts in this direction should focus on both bracts and sepals. Further studies on the development, structure and position of internal mucilage-secreting tissues in bromeliad reproductive organs are strongly encouraged, as they could provide insights into the taxonomy, evolution and ecology of the group.
(c) *experimental evidence on the modes of water acquisition by the reproductive axis.* While an extensive amount of research has been done on strategies to obtain and use water by the vegetative shoot of bromeliads, virtually nothing is known about the modes of acquisition of water and nutrients in the reproductive axis of bromeliads that lack functional roots or depend heavily on stored water.
(d) *relationship between mucilaginous secretions and water balance.* Due to the hygroscopic characteristics of mucilage, the secretion produced by the trichomes, the ‘mucilaginous tissue’ and other internal mucilage-secreting structures could be associated with mechanisms of water acquisition and allocation in the ­inflorescences of some bromeliads.
(e) *relationships between lipophilic secretions, protection against desiccation, high-radiance, herbivory and indirect nutrient supply.* The ability of lipophilic secretions to avoid excessive transpiration, provide insulation and protect against herbivores needs further evaluation. Likewise, the involvement of these secretions in an indirect input of nutrients (via insect carcases trapped in the secretion; Monteiro and Macedo 2014; Ballego-Campos *et al.* 2023) is an interesting perspective that should not be neglected.

## Conclusions

Secretion in inflorescences of Tillandsioideae is likely to be a widespread phenomenon. The secretory activity is carried out by peltate trichomes in the adaxial surface of floral bracts during their expansion and can result in either hydrophilic or mixed (e.g. with both hydrophilic and lipophilic contents) exudates. At least in some members of *Vriesea* (*V*. aff. *bituminosa* and *V. fenestralis*), the secretion is resinous (Ballego-Campos *et al.* 2023). These distinct exudates are likely to engage in colleter-like roles, such as protection against desiccation, high-radiation and herbivores. An indirect association with nutrient supply via trapped insect carcasses and pollination syndrome is also suggested.

Bract anatomy revealed the presence of a distinct ‘mucilaginous tissue’ in the midrib portion. This feature is largely unknown in bromeliads, and we present evidence of a widespread occurrence within subtribe Vrieseinae, comprising a putative synapomorphy.

## Data Availability

All data are summarized in the article. Further details are available upon reasonable request to the corresponding author.
